# Effect of the *COMT* Val158Met genotype on lateral prefrontal activations in young children

**DOI:** 10.1111/desc.12649

**Published:** 2018-01-04

**Authors:** Yusuke Moriguchi, Ikuko Shinohara

**Affiliations:** ^1^ Graduate School of Education Kyoto University Yoshidahoncho Kyoto Japan; ^2^ Department of School Education Joetsu University of Education Yamayashikicho Joetsu Japan; ^3^ Japan Science and Technology Agency Kawaguchi Saitama Japan; ^4^ National Institute for Educational Policy Research of Japan Chiyodku Tokyo Japan

## Abstract

Low executive function (EF) during early childhood is a major risk factor for developmental delay, academic failure, and social withdrawal. Susceptible genes may affect the molecular and biological mechanisms underpinning EF. More specifically, genes associated with the regulation of prefrontal dopamine may modulate the response of prefrontal neurons during executive control. Several studies with adults and older children have shown that variants of the catechol‐O‐methyltransferase (*COMT*) gene are associated with behavioral performance and prefrontal activations in EF tasks. However, the effect of the *COMT* genotype on prefrontal activations during EF tasks on young children is still unknown. The present study examined whether a common functional polymorphism (Val158Met) in the *COMT* gene was associated with prefrontal activations and cognitive shifting in 3‐ to 6‐year‐old children. The study revealed that, compared with children with at least one Met allele (Met/Met and Met/Val), children who were Val homozygous (i) were more able to flexibly switch rules in cognitive shifting tasks and (ii) exhibited increased activations in lateral prefrontal regions during these tasks. This is the first evidence that demonstrates the relationship between a gene polymorphism and prefrontal activations in young children. It also indicates that *COMT* Val homozygosity may be advantageous for cognitive shifting and prefrontal functions, at least during early childhood, and children who possess this variant may have a lower risk of developing future cognitive and social development issues.

**RESEARCH HIGHLIGHTS**

- Variants of the catechol‐O‐methyltransferase (*COMT*) gene are associated with the regulatory mechanism of prefrontal dopamine that may modulate prefrontal function during executive control.
- We examined whether a common functional polymorphism (Val158Met) in the *COMT* gene associated with prefrontal activations and cognitive shifting in 3‐ to 6‐year‐old Japanese children.
- Older rather than younger children who were Val homozygous appeared to more flexibly switch rules compared to peers with at least one Met allele.
- Children who were Val homozygous exhibited stronger activations in lateral prefrontal regions than children with at least one Met allele during cognitive shifting tasks.

## 1 INTRODUCTION

Executive function (EF) is the ability to control both thoughts and actions and consists of several cognitive processes, such as (i) updating information in working memory, (ii) inhibiting dominant and/or inappropriate responses, and (iii) flexibly switching between one task and the other (i.e., cognitive shifting) (Garon, Bryson, & Smith, 2008; Miyake & Friedman, 2012). Recently, research has shown that the quality of EF during early childhood is a predictor for several aspects of adolescent and adult life, including academic achievement, peer‐to‐peer relationships, and socioeconomic and health status (Moffitt et al., 2011). Therefore, identifying factors contributing to individual differences in EF during early childhood has become one of the most important research topics in developmental and psychological science.

Although recent studies investigating individual differences in the EF of children have focused on environmental factors, such as parenting and socioeconomic status (SES) (Bernier, Carlson, & Whipple, 2010; Diamond, Barnett, Thomas, & Munro, 2007; Noble, Norman, & Farah, 2005; Wong et al., 2010), the role of genetic and biological factors has also received a great deal of attention recently (Blair et al., 2015; Diamond, Briand, Fossella, & Gehlbach, 2004; Weikum et al., 2013). Behavioral genetic research using a twin‐study design suggests that genetic influences contributing to individual differences in EF may be affected by age (Fujisawa, Todo, & Ando, 2017). In more detail, genetic effects on individual differences in EF amongst adults can be stronger compared to younger populations (Friedman, Miyake, Robinson, & Hewitt, 2011; Friedman et al., 2008), despite a strong shared and non‐shared environmental influence on individual differences in EF among preschool children (Fujisawa et al., 2017). This latter study suggests that genetic factors begin to contribute to individual differences in EF during early childhood.

**Figure 1:**
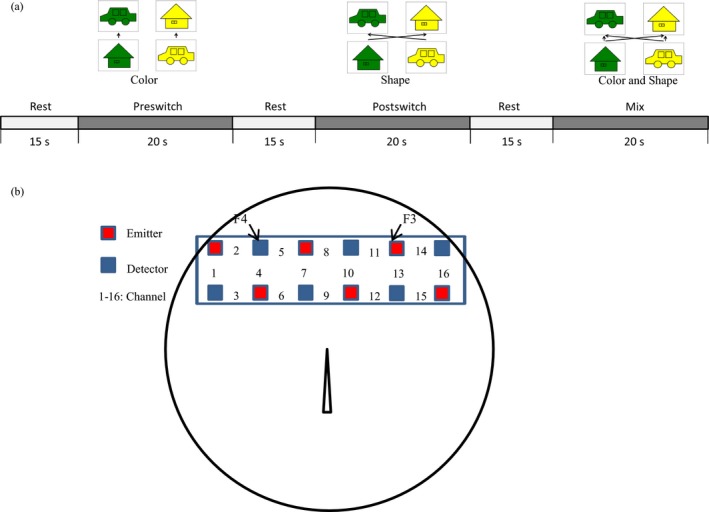
Experimental settings. (a) Experimental sequence. (b) The NIRS probe was attached to the lateral prefrontal area. Each channel consisted of one emitter optode and one detector optode. The regions of interest were located near F3/4, corresponding to channels 2, 4, and 5 and channels 11, 13, and 14, respectively

It has been shown that lateral prefrontal regions contribute to EF in adult populations (Miller & Cohen, 2001). In the developmental literature, EF development is related to activity in the neural network including prefrontal cortex, parietal cortex and subcortical regions during childhood (Bunge, Dudukovic, Thomason, Vaidya, & Gabrieli, 2002; Moriguchi, 2017; Morton, Bosma, & Ansari, 2009). In terms of prefrontal cortex, recent neuroimaging studies show that infants and children recruit the lateral prefrontal cortex during EF tasks, including working memory and cognitive shifting (Baird et al., 2002; Moriguchi & Hiraki, 2009, 2011; Tsujimoto, Yamamoto, Kawaguchi, Koizumi, & Sawaguchi, 2004). For example, by using functional near‐infrared spectroscopy (fNIRS) to measure brain activity in children, Moriguchi and Hiraki (2009) found that the development of cognitive shifting during the Dimensional Change Card Sort (DCCS, Figure 1a) task associated with increased activations within lateral prefrontal cortex. Lateral prefrontal cortex is clearly important for EF, which in turn suggests that there are specific features within this area of the brain that contribute to individual differences in children's EF.

In this regard, dopamine is an important neurotransmitter required for functions within lateral prefrontal cortex. Catechol‐O‐methyltransferase (*COMT*) is the major enzyme that contributes to the degradation of released dopamine within this region of the brain (Karoum, Chrapusta, & Egan, 1994; Weinshilboum, Otterness, & Szumlanski, 1999). Several studies have shown that variants of the *COMT* gene are associated with behavioral performance and prefrontal activation in EF tasks. These include a functional missense mutation at codon 158 that results in a substitution of methionine (Met) for valine (Val). The substitution of Met for Val results in a three‐ to fourfold reduction in enzyme activity, leading to prolonged dopamine action in the prefrontal cortex (Chen et al., 2004). Thus, compared with Val homozygous adults, individuals with the Met allele of the *COMT* gene show better performance on tasks involving EF, such as the Wisconsin Card Sorting Test (WCST) (Barnett, Jones, Robbins, & Müller, 2007; Egan et al., 2001).

The WCST includes several complex cognitive processes, such as cognitive shifting, working memory, and error detection. Therefore, subsequent studies have focused on specific components of EF. Research using working memory tasks reported similar results to the WCST, where participants with the Met allele of the *COMT* gene showed better performance compared to those who were Val homozygous (Bruder et al., 2005; Egan et al., 2001). On the other hand, an opposite effect was observed during research using cognitive shifting tasks. Participants who were Val homozygous showed more flexible switching compared to those with the Met allele (Bilder, Volavka, Lachman, & Grace, 2004; Colzato, van den Wildenberg, & Hommel, 2014; Colzato, Waszak, Nieuwenhuis, Posthuma, & Hommel, 2010). Researchers suggest that the high level of the dopamine in prefrontal regions (as found in people with the Met allele) is beneficial for cognitive stability (working memory), but may be disadvantageous for cognitive flexibility (Cools, 2006).

The relationship between the *COMT* gene, EF, and the prefrontal cortex is complex. Adult brain imaging studies repeatedly show that the Met carriers who perform better on working memory tasks show less (i.e., more efficient) lateral prefrontal activation than worse performing Val carriers (Caldú et al., 2007; Egan et al., 2001). Conversely, several other studies examining other tasks, such as inhibitory control, report that increased prefrontal neural activity is found in individuals with the Met allele who also performed these tasks better (Congdon, Constable, Lesch, & Canli, 2009; Winterer et al., 2006). This discrepancy may be due to the association of behavioral performance and activation in the prefrontal regions (Jaspar et al., 2014). For example, there are different neural signatures for EF efficiency: in working memory studies, superior performance is associated with decreased activity within the prefrontal regions, whereas superior performance involving inhibition tasks is associated with increased prefrontal activity.

However, there is limited evidence examining the effect of the *COMT* gene in young children. Blair et al. (2015) reported that Val homozygous preschool children performed better on EF tasks than Met carriers, especially when children had adverse life experiences. However, Blair et al. reached this conclusion by calculating composite scores on several EF tasks, including working memory and cognitive shifting. Moreover, there is an absence of evidence to show if, and subsequently how, the *COMT* gene is associated with EF and prefrontal activation in young children. To address these issues, the present study examined prefrontal activation using fNIRS in 3‐ to 6‐year‐old children performing the DCCS task. The relationship between task performance, prefrontal activation, and the *COMT* Val158Met genotype was assessed. As socioeconomic status (SES) is correlated with EF (Noble et al., 2005), we included SES as a covariate.

Previous research has shown that Val homozygosity is associated with better performance on tasks involving cognitive shifting among the adult population (Colzato et al., 2014; Colzato et al., 2010). In addition, superior performance during the DCCS task is associated with increased activation within lateral prefrontal regions of young children (Moriguchi & Hiraki, 2009). Thus, we predicted that young children who were Val homozygotes would (i) perform better in an EF task involving cognitive shifting (i.e., the DCCS) and (ii) exhibit increased lateral prefrontal activation compared to Met carriers.

## 2 METHODS

### 2.1 Participants

Participants included 81 3‐ to 6‐year‐old children. Previous studies investigating cognitive and brain development have shown that the *COMT* genotype interacts with children's age (Dumontheil et al., 2011; Sugiura et al., 2017). Therefore, children were split into younger (*N* = 42; males = 20; *M* age = 50.8 months, S*D* = 5.5, range = 42–59 months) and older (*N* = 39; males = 19; *M* age = 68.9 months, S*D* = 5.4, range = 61–77 months) groups and results are reported separately. All children were recruited from nursery schools in Osaka, Japan and did not have any known developmental abnormalities. An additional 12 children were tested but excluded from the final analyses because of their refusal to wear the NIRS probe (*n* = 7) and experimental error (*n* = 3). Moreover, two parents did not report their SES, and these children (*n* = 2) were also excluded from the analyses. Informed consent was obtained from all parents prior to their child's involvement in the study, which was conducted in accordance with the principles of the Declaration of Helsinki and approved by the local ethics committee.

### 2.2 Genotyping

DNA extraction, polymerase chain reaction (PCR), and single nucleotide polymorphism (SNP) typing were carried out at the J‐Bio 21 Center (Nippon Steel & Sumikin Eco‐tech Corp., Tokyo, Japan). Cheek‐swab samples (with parental and child consent) were collected in accordance with the manufacturer's instructions, using a sterile omni‐swab (DNA Extraction Kit, J‐Bio21 Center, Ibaraki, Japan). A quenching probe (QP) method was conducted to genotype the *COMT* Val/Met (rs4680) SNP using i‐density (Arkray, Inc., Kyoto, Japan). Briefly, a DNA fragment containing the target SNP was amplified by PCR, after which the fragment was hybridized with a QP with a complementary sequence. Next, the fluorescence intensity of the QP was measured at different temperatures. The fluorescence intensity depends on QP dissociation from the amplicon, and QP dissociation is affected by temperature. Thus, by detecting the fluorescence intensity, it is possible to detect genetic differences as small as a single‐base substitution in the sequence of a DNA fragment (Isegawa et al., 2010). Each sample was analyzed twice, and the results of the analyses were in agreement across all samples.

### 2.3 Socioeconomic status

Maternal education and family income were used as indices of social environment. In terms of maternal education, each parent's education level was assigned a value from 1 to 5 (less than high school 1, high school 2, some college 3, undergraduate degree 4, graduate level 5). Five mothers had graduated from junior high school and 26 mothers from high school. Thirty‐eight mothers had graduated from a career college, and 12 mothers had an undergraduate degree. No mothers were scored as graduate level 5. In addition, a self‐reported measure of family income was obtained in 12 possible categories: 0–\1,000,000 (*n* = 3), \1,000,001–\2,000,000 (*n* = 10), \2,000,001–\3,000,000 (*n* = 5), \3,000,001–\4,000,000 (*n* = 15), \4,000,001–\5,000,000 (*n* = 19), \5,000,001–\6,000,000 (*n* = 5), \6,000,001–\7,000,000 (*n* = 6), \7,000,001–\8,000,000 (*n* = 7), \8,000,001–\9,000,000 (*n* = 3), \9,000,001–\10,000,000 (*n* = 5), \10,000,001–\15,000,000 (*n* = 1), and > \15,000,001 (*n* = 2). The mother's education and family income were converted to *z*‐scores and averaged to create the total SES scores.

### 2.4 Behavioral tasks

To assess effects on behavior, children were asked to perform the DCCS task. We used a modified version of the NIH tool box (Zelazo et al., 2013) that adapted the material and procedures to allow for brain activity acquisition during assessment, as described in previous studies using NIRS (Moriguchi, Sakata, Ishibashi, & Ishikawa, 2015). Laminated cards (3.5 cm × 7.0 cm) that had two dimensions (shape and color) were used as stimuli (Figure 1a). The task included target cards and test cards: target cards matched test cards in one dimension but did not match in the other dimension (e.g., target cards of a yellow house, or a green car, and test cards of green houses or yellow cars). The experiments included three different pairs of target and test cards. At each session, a different tray with a different set of cards was used.

Children performed three consecutive test sessions. One session consisted of a rest phase (15 s), pre‐switch phase (20 s), second rest phase (15 s), post‐switch phase (20 s), third rest phase (15 s), and mixed phase (20 s). During the pre‐switch phase, children were given instructions regarding the first rule (e.g., “This is a shape game. All the cars go here, and all the houses go there”). During the post‐switch phase, they were asked to sort the cards according to the second rule (i.e., color). Finally, during the mixed phase, children performed the task after they were given instructions regarding the rule (e.g., “This is a mixed game. In this game, you will use both shape and color rules.”). In each phase, children were given a rule for each trial (e.g., color). The rule order (e.g., color first) during these pre‐switch and post‐switch phases was held constant across the three sessions for each child, but the rule order was counterbalanced across children. During each phase, children participated in an average of eight trials. The number of the trials was based on the duration for each phase and it did not significantly correlate with the percent correct (*r*(81) = −0.130, *p* = .243). The rule order during the mixed phase was fixed: POST (the rules for the post‐switch phase), POST, PRE (the rules for the pre‐switch phase), POST, POST, PRE, POST, POST. The number of the PRE trials was not the same as the number of the POST trials based on a previous study (Zelazo et al., 2013).

The dependent measures were the percentage of (i) correct responses and (ii) successful switching. We calculated the percentage of successful shifting as a measure to index total performance because the pre‐switch and post‐switch phases are easy for older children. Children needed to switch rules between the pre‐switch and post‐switch phases (one switch). The pass criterion was 90% correct in the pre‐switch and post‐switch phases (Towse, Redbond, Houston‐Price, & Cook, 2000). Moreover, children needed to switch the rules during the mixed phase four times (four switches). Thus, we calculated the percentage of successful switching out of five switches.

### 2.5 NIRS recordings

NIRS measurements were performed during the DCCS tasks. A multichannel NIRS unit operating at wavelengths of 770 and 840 nm (OEG‐16; Spectratech Inc., Tokyo, Japan) was used to measure temporal changes in the concentrations of oxygenated hemoglobin (oxy‐Hb) and deoxygenated hemoglobin (deoxy‐Hb). The NIRS probes included 12 optodes, which constituted 16 channels, and the probes were placed on the lateral prefrontal areas of each hemisphere. Each channel consisted of one emitter optode and one detector optode located 3 cm apart. The temporal resolution at each channel was approximately 666 ms. These probes were placed on the lateral prefrontal areas of each hemisphere.

The region of interest (ROI) was located near F3/4 of the International 10/20 system, corresponding to Brodmann areas (BA) 9/46. This decision was based on previous studies that show these areas to be activated during DCCS tests (Moriguchi et al., 2015; Morton et al., 2009). The spatial resolution of the NIRS is relatively low, and therefore, channels 2, 4, and 5 and channels 11, 13, and 14 were defined as corresponding to the right and left lateral prefrontal regions, respectively (Figure 1b).

We measured changes in oxy‐Hb and deoxy‐Hb in lateral prefrontal areas during the rest phases and each of the task phases. The average changes in oxy‐Hb and deoxy‐Hb during the rest and the task phases were calculated for each channel (channels 11, 13, and 14 and channels 2, 4, and 5) in each subject.

We separated the NIRS signal into functional (i.e., brain activations) and systematic (i.e., physiological noise) components. Recently, it has been shown that the NIRS signal can be contaminated by physiological activities other than cerebral function, such as cardiac pulsation, respiration, and body motion (Huppert, Diamond, Franceschini, & Boas, 2009). Several techniques have been proposed to remove such physiological artifacts. Our approach involved separating functional signals from systemic signals (e.g., physiological activities), based on a negative or positive linear relationship between oxy‐Hb and deoxy‐Hb changes (Yamada, Umeyama, & Matsuda, 2012). This method utilizes the known characteristics of NIRS signals in that oxy‐Hb and deoxy‐Hb negatively correlate with cerebral function, whereas oxy‐Hb and deoxy‐Hb are positively correlated with systemic function. We therefore used this technique to separate signals into functional and systemic components, using only the former, for subsequent analyses.

## 3 RESULTS

### 3.1 *COMT* genotyping

The *COMT* genotypes (*n* = 6 Met/Met, *n* = 37 Met/Val, *n* = 38 Val/Val) were in Hardy–Weinberg equilibrium, χ^2^(1, *n* = 81) = 0.626, *p* = .731. Given that the number of participants with the Met/Met genotypes was small, we combined the Met/Met genotypes and Met/Val genotypes into the ≥1 Met allele group. We compared performance of the ≥1 Met allele group with those of Val/Val homozygotes (Val/Val group). This grouping has been used in previous studies with Asian participants where the number of individuals with the Met/Met genotype was small (Wang et al., 2013).

### 3.2 Behavioral results

**Figure 2:**
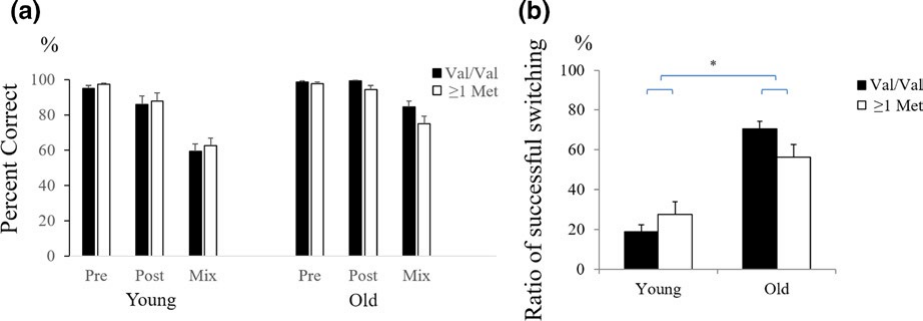
Behavioral results. (a) Percent correct and (b) ratio of successful switching

First, we recorded the number of correct responses in each phase. We then calculated the percentage of correct responses during the pre‐switch, post‐switch, and mixed phases. With regard to the percentage of correct responses, both younger and older children showed near‐perfect performance for card sorting during the pre‐switch phase, and accuracy during the post‐switch phase was lower than during the pre‐switch phase in younger children. Moreover, children showed difficulty performing during the mixed phase (Figure 2a).

The percentage of correct responses was analyzed using an age (old vs. young) × *COMT* genotype (Val/Val vs. at least one Met allele) × phase (pre‐switch vs. post‐switch vs. mix) mixed ANCOVA with SES (high vs. low) as a covariate. We observed a significant main effect of age [*F*(1, 76) = 15.547, *p* < .001, η^2^ = 0.15] and phase [*F*(2, 152) = 105.720, *p* < .001, η^2^ = 0.55], qualified by a significant interaction between these two variables [*F*(2, 76) = 9.454, *p* < .001, η^2^ = 0.10]. Post‐hoc analyses of this interaction (corrected using the Bonferroni method) revealed that older children performed better than younger children in the post‐switch and mixed phases (*p* < .029), but not in the pre‐switch phase (*p* > .345). No other significant results were observed.

Given that good performance in the pre‐switch phase is a prerequisite for being able to conclude that performance in the post‐switch phases reflected switching, we examined whether *COMT* genotype affected the percentage of correct responses in the post‐switch and the mixed phases in the DCCS tasks for children who performed more than 90% in the pre‐switch phases. We conducted an age (old vs. young) × *COMT* genotype (Val/Val vs. at least one Met allele) × phase (post‐switch vs. mix) mixed ANCOVA with SES (high vs. low) as a covariate. Again, we found no significant main effects of *COMT* genotype and significant interactions between *COMT* genotype and other variables (*ps* > .100).

Next, the percentage of successful shifting was analyzed using an age (old vs. young) × *COMT* genotype (Val/Val vs. at least one Met allele) ANCOVA, with SES as a covariate (Figure 2b). Age was found to be a main effect [*F*(1, 76) = 48.432, *p* < .001, η^2^ = 0.39], and a significant interaction was identified between age and *COMT* genotype [*F*(1, 76) = 5.110, *p* = .027, η^2^ = 0.06]. Post‐hoc analyses of the interaction between age and *COMT* genotype (Bonferroni corrected) revealed that the *COMT* genotype had no effect on successful shifting in younger children (*p* = .434). However, the Val/Val group were better at switching than the ≥1 Met allele group in older children (*p* = .020). No other significant effects were found.

These results are consistent with a previous study suggesting that the effects of the *COMT* gene were observed in 4‐ and 5‐year‐old children, but not in 3‐year‐old children (Blair et al., 2015).

### 3.3 NIRS results

Next, we examined whether the lateral prefrontal regions were activated during the DCCS task. The results for the oxy‐Hb and deoxy‐Hb measurements were consistent after separating the NIRS signal into functional and systematic components, and we therefore report the oxy‐Hb results. The significance of the possible difference between changes in oxy‐Hb for both the rest phase and task phase was determined by a two‐tailed Student's *t* test for each channel. The behavioral data showed that the percentage of successful shifting was associated with genetic effects, and we calculated brain measures to index successful shifting. Specifically, we combined each rest phase into an aggregated rest phase, and each task phase (pre‐switch, post‐switch, and mixed phases) into an aggregated task phase. We compared the aggregated task phase to the rest phase using a multiple comparison test and applied a 0.004 (0.05/12) alpha level of significance (two groups and six channels).

**Figure 3:**
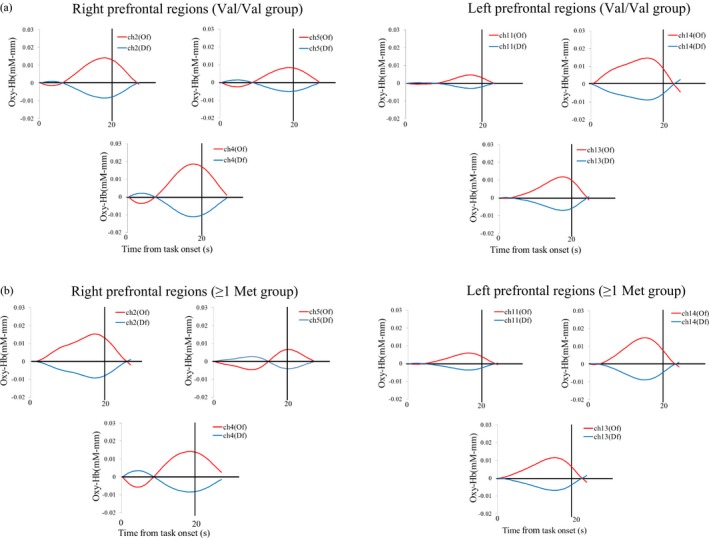
Temporal changes in the oxy‐Hb (red) and deoxy‐Hb (blue) concentration within the right (channels 2, 4, and 5) and left (channels 11, 13, and 14) lateral prefrontal areas during performance of the DCCS tasks. The brain measures index was calculated as the percentage of successful shifting by combining each task phase (pre‐switch, post‐switch, and mixed) into an aggregated task phase. Aggregated task phases were compared with the rest phase. Group mean data from children in the (a) Val/Val group and (b) ≥1 Met allele group are shown

We analyzed separately lateral prefrontal activations in the Val/Val and ≥1 Met allele groups. Children in the Val/Val group had significantly activated right (Channel 2, 4, and 5) and left (channel 14) prefrontal regions during the task phases compared to the rest phase (Student's *t* test, *p* < .004; Figure 3a). Children in the ≥1 Met allele group exhibited significant activation in both the right (Channel 2 and 4) and left (channel 14) prefrontal regions during the task phases, compared to during the rest phase (Student's *t* test, *p* < .004; Figure 3b).

**Figure 4:**
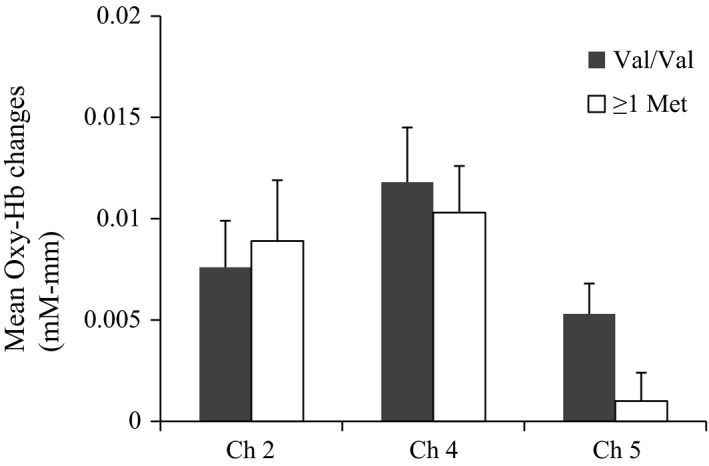
Mean oxy‐Hb changes within the right lateral prefrontal areas (channels 2, 4, and 5) in the Val/Val group and the ≥1 Met allele group during performance of DCCS tasks. Error bars indicate standard error

Next, we directly compared activation in the lateral prefrontal regions of the Val/Val and ≥1 Met allele groups. As the prior analyses indicated some differences in the right prefrontal regions (i.e., more channels for the Val/Val group), we therefore focused on the right prefrontal regions. To assess any differences, we conducted a MANCOVA with mean changes in oxy‐Hb (channels 2, 4, and 5) as the dependent variable, age (old vs. young) and *COMT* genotype (Val/Val vs. at least one Met allele) as the independent variables, and SES as the covariate (Figure 4). The results of this analysis revealed that *COMT* genotype had a significant main effect on channel 5 [*F*(1, 76) = 4.370, *p* = .040, η^2^ = 0.05], indicating that children in the Val/Val group showed stronger activations at channel 5 than those in the ≥1 Met allele group. No other significant results were obtained.

## 4 DISCUSSION

The present study provides the first evidence for a relationship between executive function (EF), gene polymorphism, and the prefrontal cortex in young children. Research on adult subjects suggests that *COMT* genotypes may affect the molecular and biological mechanisms of EF. However, it was unclear how these genotypes affected individual differences in EF in young children. Our findings show that, compared with children with at least one Met allele, Val homozygous Japanese children (i) showed superior performance and (ii) showed increased activation in at least one localized region of prefrontal cortex during the DCCS tasks.

Although adults of European descent with the Met allele of the *COMT* gene show better performance in working memory tasks than those who are Val homozygous (Egan et al., 2001), research using cognitive shifting tasks has shown that Val homozygotes outperform individuals homozygous for Met (Colzato et al., 2014; Colzato et al., 2010). Our results are consistent with the latter study, showing superior performance by Val homozygous young Japanese children.

However, the relationship between the *COMT* gene, EF, and prefrontal activations should be interpreted with caution. Adult Met carriers who perform better in working memory tasks show weaker lateral prefrontal activations than Val homozygote carriers who perform worse (Barnett et al., 2007; Egan et al., 2001). On the other hand, Met carriers who perform better in inhibitory control tasks compared to Val carriers show increased neural activity (Congdon et al., 2009; Winterer et al., 2006). It is possible that efficiency in some EF tasks is associated with increased prefrontal activity (i.e., inhibition), whilst other tasks are associated with decreased prefrontal activity (i.e., working memory) (Jaspar et al., 2014). Regarding young children, superior performance has been associated with increased prefrontal activity during DCCS tasks (Moriguchi & Hiraki, 2009; Moriguchi et al., 2015). Therefore, it seems that Japanese children, at least, who are Val homozygotes show better cognitive shifting and more prefrontal activity than Met carriers, although we have to note that what increased and decreased activities in prefrontal cortex reflect may differ across children and adults, because lateral prefrontal cortex continues to develop during childhood (Gogtay et al., 2004).

We also found that the effects of *COMT* genotype on behavioral performance were different from those for prefrontal activations. More specifically, the effects on behavioral performance were observed only in older children, whereas the effects of *COMT* genotype on brain activity were observed regardless of age. These results indicate that the effects associated with *COMT* genotype are expressed earlier in the brain than in behavior. Such results are not necessarily surprising after acknowledging that activations in prefrontal cortex are a more sensitive (and direct) measure of the effects of dopamine and *COMT* genotypes. Others, too, have found evidence of effects (advances as well as declines) earlier in the brain than on behavioral measures (Bookheimer et al., 2000; Friederici, Friedrich, & Christophe, 2007). Moreover, one adult fMRI study reported that, despite significant differences in prefrontal and temporal activation, there were no significant effects between different *COMT* genotype carriers on 19 cognitive measures, including EF measures (Dennis et al., 2009), although other studies have found differences in EF performances by *COMT* genotype.

One might argue that children performed the pre‐switch phases well because they did not include any switching. Therefore, including the NIRS results in an examination of the pre‐switch phases may add noise to the neural indices. However, to create the neural indices that corresponded to behavioral performance, our behavioral analyses included the pre‐switch, post‐switch, and mixed phases. Moreover, previous studies have shown that activations in the prefrontal cortex during both pre‐switch and post‐switch phases are important for rule switching during DCCS tasks (Moriguchi & Hiraki, 2009, 2011). In addition to NIRS studies, event‐related potential (ERP) studies have confirmed that neural activation during the pre‐switch and post‐switch phases contributed to the performance in the post‐switch phases (Espinet, Anderson, & Zelazo, 2012, 2013). Thus, prefrontal activations in the pre‐switch phases may contribute to flexible switching during DCCS tasks.

Our results show that children who were Val homozygotes outperformed those with at least one Met allele. However, genetic effects may be more pronounced in this study, as previous studies have reported a Val‐associated advantage in the Asian population. Indeed, it has been proposed that the function of the *COMT* genotype may vary across different cultures. Research in China has shown that Val carriers demonstrated superior performance during several cognitive tasks, including executive function, when compared to Met homozygotes (Wang et al., 2013; Yeh, Chang, Hu, Yeh, & Lin, 2009). Therefore, future studies should assess whether the observed genetic differences in young children associated with cognitive shifting and prefrontal activations are affected by ethnic grouping.

The results of our study advance understanding of potential individual differences in a child's EF. EF during early childhood is a predictor for academic achievement and social status during adolescence and adulthood (Moffitt et al., 2011), and several studies attempt to identify and understand genetic and environmental effects on individual differences in EF during early childhood (Blair et al., 2015; Diamond et al., 2007; Diamond et al., 2004; Noble et al., 2005). Our study clearly shows that genetic and biological factors play a role. Young children who are homozygous for the Val allele were more likely to show better cognitive shifting than children with at least one Met allele. This, in turn, may decrease the risk of future impairment due to reduced cognitive and social development and this proposal is consistent with a previous study (Blair et al., 2015). In contrast, Met carriers may be at risk of developing impaired cognitive shifting during early childhood. This may be because Met carriers are more sensitive to environmental factors, such as early adverse experiences, when compared to Val homozygotes (Diamond, 2011). Nevertheless, we have assessed only children's cognitive shifting in this study, and different results may be obtained when working memory tasks are examined.

Finally, the *COMT* gene is almost certainly not the only gene that has alleles that contribute to EF and prefrontal activations in young children. Future research should be undertaken to identify other susceptible genes that may affect the molecular and biological mechanisms that underpin EF. Moreover, prefrontal regions are not the only brain regions that contribute to EF. Indeed, recent brain imaging studies have shown that the brain network including lateral prefrontal cortex, parietal cortex, as well as subcortical regions and their interaction play an important role in the development of EF (Moriguchi, 2017; Morton et al., 2009). Future research should assess how the brain network contributes to the development of EF during childhood.
